# Aberrant Gene Regulation in Obstetric, Gynecologic, and Reproductive Diseases

**DOI:** 10.1155/2015/187691

**Published:** 2015-08-23

**Authors:** Shi-Wen Jiang, Brian Brost, Dan Zhang, Chun-E Ren

**Affiliations:** ^1^Department of Biomedical Science, Mercer University School of Medicine, Savannah, GA 31405, USA; ^2^Department of Obstetrics and Gynecology, The Second Affiliated Hospital of Wenzhou Medical University, Wenzhou, Zhejiang 325027, China; ^3^Anderson Cancer Institute, Memorial Health Hospital, Savannah, GA 31404, USA; ^4^Department of Obstetrics and Gynecology, Mayo Clinic, Mayo Medical School, Rochester, MN 55905, USA; ^5^Department of Reproductive Endocrinology, Women's Hospital, Zhejiang University School of Medicine, Hangzhou 310006, China; ^6^Department of Obstetrics and Gynecology, Weifang Medical University, Weifang, Shandong 201031, China; ^7^Center for Reproductive Medicine, Affiliated Hospital of Weifang Medical University, Weifang, Shandong 261031, China

Abnormal gene expression is often detected in various tissues/organs of patients with gynecologic and reproductive diseases. Research efforts on the regulatory mechanisms and molecular pathways will lead to a better understanding of the pathophysiological process of these diseases. Identification of the key factors and their roles in gynecologic diseases may also facilitate the development of novel diagnostic markers and therapeutic models. This special issue compiles excellent original and review articles covering gene expression and function in the obstetric, gynecologic, and reproductive disorders.

Two articles are from the field of reproductive endocrinology. Using a cell coculture model, X. Liu et al. characterized the paracrine regulation of steroidogenesis in theca cells by granulosa cells derived from mouse preantral follicles. It was found that granulosa cells were able to promote steroidogenesis and responsiveness to luteinizing hormone in theca cells. M. Rahnama et al. investigated the changes of osteocalcin gene expression in postmenopausal women treated with Hormone Replacement Therapy (HRT) and observed a correlation between osteocalcin gene expression in areas of oral cavity and bone metabolism in these women.

H. Ge et al. report that mitochondrial uncoupling protein 2 (UCP2) is expressed in cultured human cumulus cells and may contribute to the process of ROS production, apoptosis, and steroidogenesis, suggesting that UCP2 may be involved in the regulation of follicle development and oocyte maturation. Y. Zou et al. present data showing that Decorin, a decidua-derived TGF-binding proteoglycan, inhibits the proliferation, migration, and invasion of human trophoblast cells. In the same in vitro culture, Decorin can also promote cell apoptosis.

Noncoding RNAs and their epigenetic actions on gene expression are a hot research topic. P. Laudanski et al. report their results on the profiling of selected microRNAs in proliferative eutopic endometrium of women with ovarian endometriosis. In a parallel study, J. Chen et al. identified several regulatory target genes of miR-183, including those for integrin *β*1, AMIGO2, VAV3, and PSEN2, in endometrial stromal cell culture. They went on to show that knockdown of miR-183 expression induced the invasiveness and inhibition of apoptosis of endometrial stromal cells.

Lysophosphatidic acid (LPA) level has been found to be significantly increased in the serum of patients with ovarian, cervical, and colon cancers. Y. Sui et al. investigated the effect of LPA on the apoptosis induced by cisplatin (DDP) in cervical cancer cell lines and the underlying changes in signaling pathways.

K. T. Woolery et al. determined whether expression of the BRCA1 185delAG mutant, BRAT, in human ovarian surface epithelial cells could promote an inflammatory phenotype. Increased cellular and secreted levels of Interleukin-1*β* (IL-1*β*) were observed following BRAT expression, providing a novel mechanism by which BRAT may be involved in ovarian cancer development.

This special issue also contains meticulously prepared reviews on the recent findings. Accumulating evidence indicates that the epithelial-mesenchymal transition (EMT) is related to the metastasis and relapse of cancer. L. Campo et al. reviewed the expression and potential roles of EMT-inducing factors in various types of gynecological cancers. M. Yuan et al. review the relationship between preimplantation exposure to two endocrine disrupting chemicals bisphenol A (BPA)/triclosan (TCS) and implantation failure. Unsolved questions and possible future studies are also discussed. WNT/b-catenin pathway participates in the morphogenesis and angiogenesis of endometrium. J. Kiewisz et al. review the involvement of this pathway in normal function as well as the carcinogenic process in human endometrium.

With these articles, we hope that the special issue would provide new and insightful research information to investigators in the field of obstetrics, gynecology, and reproductive medicine.

*Shi-Wen Jiang* *Brian Brost* *Dan Zhang* *Chun-E Ren*

